# Keystone Veterinary Medical Association

**Published:** 1897-03

**Authors:** W. L. Rhoads

**Affiliations:** Secretary


Secretary.

### KEYSTONE VETERINARY MEDICAL ASSOCIATION.

THIS Association was convened on the evening of January 11th, by President J. R. Hart, with forty-three persons present, among whom were Drs. Thomas and James B. Rayner, John R. Hart, Otto G. Noack, W. L. Rhoads, W. H. Hoskins, H. A. Christman, C. J. Marshall, H. D. Martien, and as representatives of the Milk Association, Joseph H. Miller, Secretary of the Milkmen's Exchange; George Abbott, George T. Gravenstine, John W. Scott, and a number of others.

After roll-call, the reading and approval of the minutes of the December meeting, the general order of business was suspended and George Abbott was introduced as speaker of the evening. His subject was the "Milk Question." He first spoke of the milk-supply of Philadelphia, maintaining that it was comparatively above reproach, as the reports show only about 3 per cent. of adulterated milk, which he thought was very small when we consider the vast amount consumed here annually (about 97,000,000 quarts), and the many sources from which it came. Jersey furnishes 20 per cent., New York 8 per cent.; a small amount comes from Delaware, and the remainder is furnished by Pennsylvania herds, many of which must necessarily be in not the best sanitary surroundings. Yet not one case of sickness in Philadelphia has ever been traceable to the milk-supply, and this record is not likely to be broken, as the more prominent dealers are having the herds from which they procure their supply regularly examined by competent veterinarians. He then exhibited a blank such as is used in the examinations of his herds once every three months. He thinks it would be in the line of progress if the State Veterinarian would issue certificates of health (after tuberculin-test) for cattle when applied for by owner, as these held by a few would be an incentive to all other producers who wished to be in the front rank. He then spoke of the Board of Health trying to enforce the antiquated Act of 1885, commonly known as the Pittsburg Act, relating to the production and sale of milk. This Act makes no allowance for unavoidable ignorance of facts, and holds a man liable for that which he could not possibly know, being with other pure-food enactments the only instance of the kind in our courts, and not one of the European nations will impose on any class of offenders in this way. While the Act requires 12½ per cent. of total solids (only Channel Island cattle will not fall below this limit), again it prohibits milk having a specific gravity over 1.033. The milk of Channel Islands cattle has a specific gravity above the limit, consequently all dealers would soon be in jail, except those left out through the courtesy of the milk inspectors. Regarding skim-milk, the Act requires 2¼ per cent. of fat, while only 3 per cent. of fat is required therein for whole milk; this is practically prohibiting the sale of skim milk. Dietetists are a unit as regards the comparative cost and nutrition of skim-milk. They consider it the most wholesome, healthy, as well as the cheapest animal food to be had, when its nutritive value is realized and considered. In comparison with beef and mutton the ratio of its value is as three to one in favor of skim-milk. Of the 13 per cent. of total solids in average whole milk, 4 per cent. is fat, the remainder ash, sugar, and nitrogenous products, or roast beef. The fat gives force, energy, heat, etc., but does not build up the bodily structure. Life has been sustained for eleven years on skim-milk alone; this can be accounted for by the fact that at least two-thirds of the value of whole milk still remains. The inspectors claim the use of the lactometer will shield the milkman, yet they themselves do not rely on this alone, but prosecute solely upon analytical tests, they knowing full well that part of the cream may be removed and replaced with water without affecting the lactometer's markings. He spoke of cases where the inspectors recently condemned (by the lactometer) milk which, upon analysis, showed 5 per cent. of fat, and at the same time passed milk which analysis proved to contain but little upward of 3 per cent. of fat. He thought the pure-food law should have a reservation by which the dealer would be excused if he had done all in his power to detect fraud, of which he did not know or could not by reasonable diligence ascertain.

The speaker now closed his remarks by stating that he would be glad to answer any questions. Dr. Hoskins said he thought the milk question one of vast importance, as 95 per cent. of that produced was used as a raw product, and he believed its consumption had not increased pro rata with the population in the last five years. The reply was that in 1891 and 1892, following the first attack by the Board of Health, there was a marked diminution in milk-receipts, but in 1893 and 1894 they increased about 3,000,000

quarts per year. He attributed the lack of increase since that time principally to the hard times.

It was now moved and seconded that a vote of thanks be extended Mr. Abbott for his very interesting talk; this being unanimously passed, the Association again resumed its regular order of business. Dr. Hoskins read a decision of a New York State Court in regard to dehorning, in which they held it to be a surgical operation, and the non-graduate performing it liable to a fine of fifty dollars per day while operating. This agitated the question of dehorning, which, like all progressive movements, had much to be said for and against it.

Dr. Rhoads asked what the comparative results were of ovariotomy in cattle. After some little discussion on this, Dr. James B. Rayner spoke of the removal of the uterus. This called forth remarks from Thomas B. Rayner, H. A. Christman, W. H. Hoskins, and others.

The question of a lunch to be given at the February meeting was now brought up, and, after some pro and con opinions, it was decided to give a light lunch to the Association members and its invited guests at that time.

Drs. F. S. Allen, J. T. McAnulty, and W. H. Hoskins were appointed a committee to take charge of the affair.

The meeting now adjourned to meet Tuesday evening, February 9, 1897.

At the February meeting Rev. J. D. Diedrick, of Flowertown, Montgomery county, favored the Association with a talk, giving his personal experience with the construction and use of the silo and the method of feeding ensilage, the condition of his cows, and the quality of milk produced.

At the meeting Tuesday evening, March 9th, Mr. R. A. Pearson, Assistant Chief of the Dairy Division of the Department of Agriculture, will address the Association. A generous invitation is extended to all to come to hear him.

W. L. Rhoads,
Secretary.

